# Diabetes-Induced Oxidative Stress in Endothelial Progenitor Cells May Be Sustained by a Positive Feedback Loop Involving High Mobility Group Box-1

**DOI:** 10.1155/2016/1943918

**Published:** 2015-12-21

**Authors:** Han Wu, Ran Li, Zhong-Hai Wei, Xin-Lin Zhang, Jian-Zhou Chen, Qing Dai, Jun Xie, Biao Xu

**Affiliations:** Department of Cardiology, Drown Tower Hospital, Nanjing University Medical School, Nanjing 210008, China

## Abstract

Oxidative stress is considered to be a critical factor in diabetes-induced endothelial progenitor cell (EPC) dysfunction, although the underlying mechanisms are not fully understood. In this study, we investigated the role of high mobility group box-1 (HMGB-1) in diabetes-induced oxidative stress. HMGB-1 was upregulated in both serum and bone marrow-derived monocytes from diabetic mice compared with control mice. In vitro, advanced glycation end productions (AGEs) induced, expression of HMGB-1 in EPCs and in cell culture supernatants in a dose-dependent manner. However, inhibition of oxidative stress with N-acetylcysteine (NAC) partially inhibited the induction of HMGB-1 induced by AGEs. Furthermore, p66shc expression in EPCs induced by AGEs was abrogated by incubation with glycyrrhizin (Gly), while increased superoxide dismutase (SOD) activity in cell culture supernatants was observed in the Gly treated group. Thus, HMGB-1 may play an important role in diabetes-induced oxidative stress in EPCs via a positive feedback loop involving the AGE/reactive oxygen species/HMGB-1 pathway.

## 1. Introduction

Diabetes mellitus (DM) has been widely recognized as an important modern-day disease, and cardiovascular complications are the leading causes of morbidity and mortality in DM patients. Impaired angiogenesis is thought to be a critical event contributing to the development of cardiovascular complications associated with diabetes [1]. Notably, there is agreement in the literature that endothelial progenitor cells (EPCs), the precursors of endothelial cells, may contribute to angiogenesis and endothelial repair [2, 3]. However, the number of EPCs is reduced and the function is impaired in patients with diabetes, with EPCs found to be defective in vascular repair, thus contributing to the progression of cardiovascular disease in this patients [4–6].

Oxidative stress is a major cause of various pathological processes in DM [7]. Early reports demonstrated that the number of EPCs was negatively correlated with oxidative stress, which resulted in diabetes-related EPC dysfunction [8]. Thus, oxidative stress may represent an important therapeutic target in the prevention of impaired vascular homeostasis in DM. Although discoveries made in the last decade have made it clear that oxidative stress is central to impaired angiogenesis, the endogenous mechanisms remain poorly understood.

Increasing evidence demonstrates that EPCs can be divided into two types: early and late EPCs. Early EPCs, which are generated by the culture of peripheral blood mononuclear cells in medium for approximately 4 days, exhibit higher levels of cytokine release and lower vessel growth compared with late EPCs. Interestingly, late EPCs obtained by long-term culture of early EPCs show greater vasculogenic potential and are regarded as “true EPCs” [9]. Thus, late EPCs were used to evaluate the effects of oxidative stress in this investigation.

It has been established that hyperglycemia promotes reactions between plasma proteins and glucose through a nonenzymatic process, leading to the formation of advanced glycation end productions (AGEs). AGEs are considered to be important mediators of diabetes and diabetic complications. In diabetes, AGEs accumulate in tissues at an accelerated rate and then contribute, at least in part, to the initiation and development of diabetic cardiovascular complications [10–12]. In the past decade, accumulating evidence has shown that AGEs promote oxidative stress in EPCs and then mediate EPC dysfunction in processes such as migration, tube formation, and apoptosis [13, 14].

High mobility group box-1 (HMGB-1), a nonchromosomal nuclear protein, is ubiquitously expressed in various cells including monocytes, cardiomyocytes, and endothelial cells. Once released into the serum in response to stresses such as high glucose conditions, HMGB-1 functions as a proinflammatory cytokine. Recent studies have indicated that oxidative stress promotes HMGB-1 release in various cells [15], resulting in the induction of reactive oxygen species (ROS) production in cardiomyocytes [16]. However, the role of HMGB-1 in diabetes-induced oxidative stress in late EPCs has received little attention. Thus, we hypothesized that diabetes induces the release of HMGB-1, which subsequently enhances oxidative stress in late EPCs. In the present study, HMGB-1 expression in serum and monocytes of diabetic mice was analyzed, and the role of HMGB-1 in AGE-induced oxidative stress in late EPCs was investigated in vitro.

## 2. Methods

### 2.1. Induction and Assessment of Diabetes

The experimental and feeding protocols were approved and conducted in accordance with the laws and regulations controlling experiments on live animals in China and the Asian Convention for the Protection of Vertebrate Animals used for Experimental and Other Scientific Purposes. Male C57BL/6 mice were purchased from the Model Animal Research Center of Nanjing University (China). Before injection of streptozotocin (STZ), the mice were weighed and the blood glucose was detected in a sample obtained from the tail vein using blood glucose monitoring system. Diabetes was induced in mice by consecutive intraperitoneal injections of STZ (40 mg/kg/day, Sigma-Aldrich, USA) for 5 days. After 3 days of STZ injection, blood glucose was determined as described previously. Mice with blood glucose levels >13.9 mmoL/l in three consecutive measurements were considered to be diabetic. Mice treated with citrate buffer were used as nondiabetic controls. Sixteen mice were divided into the following two groups: normal nondiabetic group (N group, *n* = 8) and diabetic group (DM group, *n* = 8). Nonfasting blood glucose and weight were measured every month until the end of the experiment.

### 2.2. Measurement of Serum HMGB-1 Levels

At the end of this investigation, the mice were anesthetized and sacrificed by decapitation, and blood was collected for serum separation. Serum HMGB-1 concentrations were determined using a mouse HMGB-1 ELISA kit (USCN Life Science, China) according to the manufacturer's instructions.

### 2.3. Isolation of Monocytes from Mouse Bone Marrow

After the mice were sacrificed, the femurs and tibias were immediately excised and flushed with cold PBS. After centrifugation at 1,400 rpm for 5 min, the collected cells were filtered and resuspended in red blood cell lysing buffer. After 5 min, the cells were centrifuged at 1,500 rpm for 5 min. After three washing steps, the monocytes were used in Western blot analyses.

### 2.4. EPC Isolation and Characterization

The protocol was approved by the Ethical Committee of Institutional Ethics Committee of Nanjing University Medical School (China). EPCs were isolated from the peripheral blood of healthy volunteers as previously described [14, 17]. Briefly, peripheral blood mononuclear cells (PBMCs) were collected by Ficoll-Paque PLUS (GE Healthcare Life Sciences, USA) density gradient centrifugation of peripheral blood. PBMCs were resuspended in endothelial cell growth medium-2 (EGM-2) (Lonza, Switzerland) composed of endothelial cell basal medium-2 (EBM-2), 5% fetal bovine serum (FBS), and growth factors containing vascular endothelial growth factor (VEGF), basic fibroblast growth factor (bFGF), epidermal growth factor (EGF), insulin-like factor-1 (IGF-1), hydrocortisone, heparin, and ascorbic acid. After 4 days in culture, medium and nonadherent cells were removed; the medium was replaced every 3 days. After approximately 3 weeks, the “cobblestone” morphology observed by microscope indicated that the cells were late EPCs. These cells (passage <5) were used in subsequent studies.

To confirm the late EPC phenotype, the cells were incubated with 5 *μ*g/mL 1,1-dioctadecyl-3,3,3,3-tetramethylindocarbocyanine-labeled low density lipoprotein (Dil-acLDL; Molecular Probes) for 2 h at 37°C and then fixed with 4% paraformaldehyde for 30 min. After being washed three times, the cells were incubated with 10 *μ*g/mL fluorescein-isothiocyanate-conjugated lectin (FITC-lectin; Sigma, USA) for 1 h at room temperature in the dark. Additionally, late EPC surface markers were detected by immunocytochemistry using FITC-CD34 (BD, USA), allophycocyanin-KDR (APC-KDR, R&D, USA), and rabbit anti-CXCR4 (Abcam, UK). Then, goat anti-rabbit Alexa Fluor 488 was used to detect the expression of CXCR4. Nuclei were stained with DAPI. The cells were observed by fluorescence microscopy (Olympus, Japan).

### 2.5. Western Blot Analysis

After being washed with cold PBS, the cells were resuspended in cell lysis buffer containing a cocktail of protease inhibitors (1 : 100; Sigma, USA). After centrifugation at 15,000 rpm for 10 min, the supernatant was collected and stored at −80°C for later use. The protein concentration of the cell lysate was detected using a BCA protein assay kit (Pierce, USA). Total cell proteins or cell supernatants were mixed with loading buffer and were heated in boiling water for 10 min. The proteins were then separated by SDS-PAGE, electrotransferred, and blotted onto polyvinylidene difluoride membranes. The membranes were blocked with 10% dried nonfat milk in 0.1% PBS-Tween-20 (PBST) for 2 h at room temperature and hybridized overnight at 4°C with anti-HMGB-1 (1 : 1,000, Bioworld Technology, USA), anti-p66shc (1 : 1,000, Santa Cruz, USA), and anti-sirt1 (1 : 500, Bioworld Technology, USA). Membranes were then washed with 0.1% PBST and incubated with appropriate secondary antibodies. The reactions were developed using enhanced chemiluminescence reagents and images were obtained by exposure to film. The bands were analyzed using BioRad Quantity One imaging software.

### 2.6. Evaluation of Intracellular ROS

Fluorescent probe 2′-7′-dichlorofluorescin diacetate (DCFH-DA; Sigma, USA) was used to measure intracellular ROS generation in late EPCs plated in 6-well plates. After different treatments, the medium was removed and the cells were washed with PBS. The cells were then incubated with 5 *μ*mol/L DCFH-DA probe in serum-free medium at 37°C for 30 min. Intracellular ROS production was detected using a fluorescence microscope (Olympus, Japan).

### 2.7. Superoxide Dismutase Activity Determination

The superoxide dismutase (SOD) activity in cell supernatants was evaluated using SOD assay kit (Jiancheng Bioengineering Research Institute, China) according to the manufacturer's instructions and was expressed as a percentage compared with the control group.

### 2.8. Statistical Analysis

Data were expressed as the means ± standard deviation (SD) and were analyzed with one-way ANOVA using SPSS 20.0 software (SPSS lnc., USA). *P* < 0.05 was considered to indicate statistical significance.

## 3. Results

### 3.1. Diabetes Induced Increased HMGB-1 in Serum and in Bone Marrow-Derived Monocytes from Diabetic Mice

**Figure 1:**
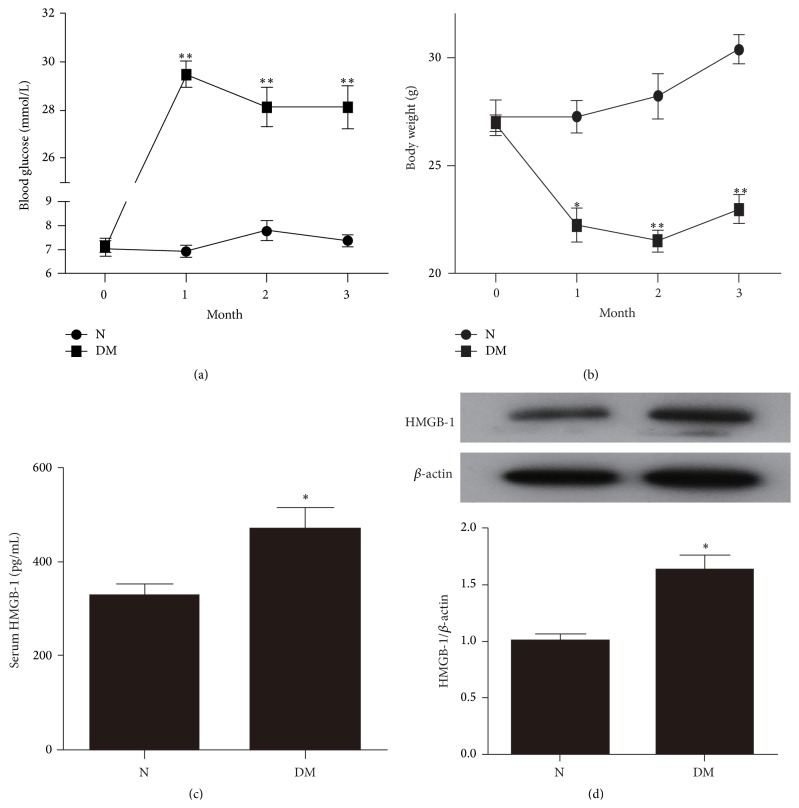
Blood glucose, body weight, and HMGB-1 expression in normal and diabetic mice. (a, b) Blood glucose and body weight in normal and diabetic mice. (c) Serum HMGB-1 levels in normal and diabetic mice. (d) Expression of HMGB-1 in bone marrow-derived monocytes in normal and diabetic mice. ^*∗*^ *P* < 0.05 and ^*∗∗*^ *P* < 0.01 DM group versus N group (*n* = 8 per group). N: normal mice. DM: diabetic mice.

As shown in Figure 1(a), diabetic mice exhibited increased blood glucose compared with normal mice; this high level was stably maintained until the mice were sacrificed. Furthermore, diabetes caused deleterious inhibition of normal weight gain during the present study (Figure 1(b)).  Figure 1(c) showed the serum HMGB-1 levels in normal and diabetic mice. STZ-induced diabetic mice showed significantly higher serum HMGB-1 levels than those of normal mice. As monocytes are the main source of serum HMGB-1, HMGB-1 expression in monocytes from normal and diabetic mice was detected by Western blot analysis showing that HMGB-1 protein levels were significantly higher in monocytes from diabetic mice than those from nondiabetic mice (Figure 1(d)).

### 3.2. AGEs Induced HMGB-1 Upregulation in Late EPCs and in Cell Culture Supernatants

**Figure 2:**
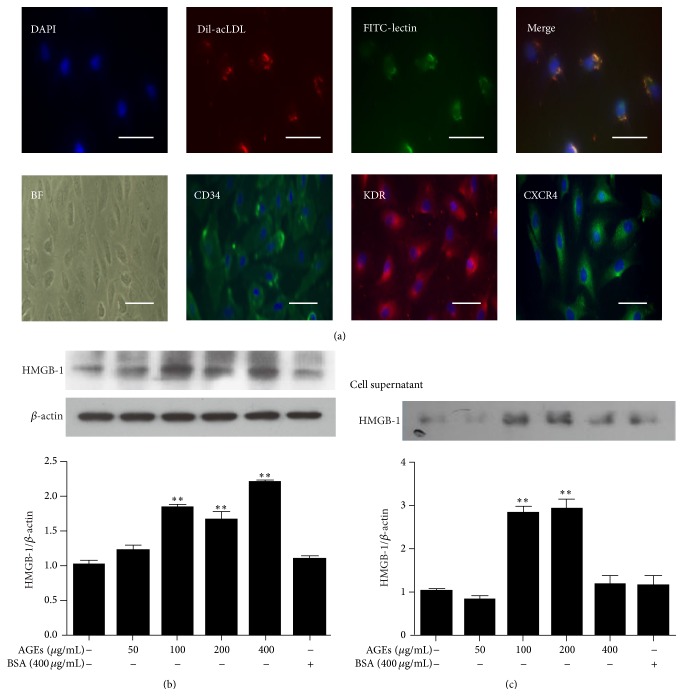
Expression of HMGB-1 in late EPCs and cell supernatants induced by AGEs at different concentrations. (a) Late EPCs were shown to endocytose Dil-acLDL and bind to lectin. Late EPCs showed a typical “cobblestone” morphology in phase-contrast inverted microscopy after approximately 3 weeks of culture and were positive for expression of CD34, KDR, and CXCR4. (b) Effect of AGEs on HMGB-1 expression in late EPCs. (c) HMGB-1 protein expression in cell culture supernatants of late EPCs exposed to AGEs. Scale bar = 20 *μ*m. ^*∗∗*^ *P* < 0.01 versus control group, *n* = 3. BF: bright field; BSA: bovine serum albumin.

More than 90% of the attached cells that were double positive for Dil-acLDL uptake and lectin staining were characterized as late EPCs. Immunophenotyping revealed that late EPCs expressed CD34 and CXCR4, the surface antigens of progenitor cells, as well as KDR, which is a characteristic surface marker of endothelial cell (Figure 2(a)). AGEs were used to establish an in vitro diabetes model to investigate the role of HMGB-1 in diabetes-induced oxidative stress. HMGB-1 levels were measured in cell culture supernatants and in late EPCs exposed to AGEs. In order to screen the optimal stimulation concentration of AGEs, late EPCs were incubated with 50–400 *μ*g/mL AGEs for 24 h, using 400 *μ*g/mL BSA as a control. As shown in Figure 2(b), AGEs induced the upregulation of HMGB-1 in late EPCs in a dose-dependent manner, and the effect reached the level of statistical significance at 100 *μ*g/mL. In addition, HMGB-1 in cell supernatants was significantly increased by exposure to AGEs at 100 *μ*g/mL or 200 *μ*g/mL (Figure 2(c)). Thus, in subsequent experiments, late EPCs were stimulated with 100 *μ*g/mL AGEs for 24 h.

### 3.3. Oxidative Stress Was Involved in AGE-Induced Increased HMGB-1 Expression

**Figure 3:**
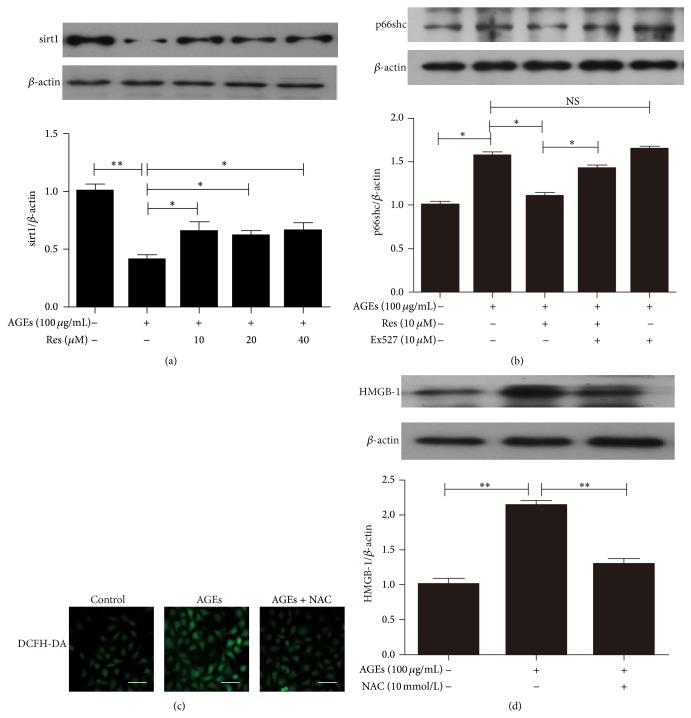
Inhibition of oxidative stress by NAC attenuated HMGB-1 expression in late EPCs. (a) AGE-induced downregulation of sirt1 was partly normalized by resveratrol. (b) Inhibition of sirt1 by Ex527 decreased p66shc expression in AGE-stimulated late EPCs. (c) DCFH-DA staining showing that pretreatment with NAC inhibited ROS production in late EPCs. (d) NAC suppressed HMGB-1 expression in AGE-stimulated late EPCs. ^*∗*^ *P* < 0.05 between the two groups and ^*∗∗*^ *P* < 0.01 between the two groups. NS: no difference between the two groups, *n* = 3. Scale bar = 25 *μ*m.

To investigate the mechanism of AGE-induced oxidative stress in late EPCs, we determined the expression of sirt1 and p66shc in late EPCs. Our results showed that AGE-stimulated EPCs displayed significantly decreased sirt1 expression, and this effect was partly reversed by pretreatment with resveratrol, an activator of sirt1 (Figure 3(a)). p66shc, a critical protein regulating cellular oxidative stress responses, is considered to be the downstream of sirt1. Increased p66shc expression in AGE-stimulated EPCs was abrogated by resveratrol (Figure 3(b)). In addition, treatment with Ex527, a sirt1 antagonist, reversed the effects of resveratrol (Figure 3(b)), suggesting that the sirt1/p66shc pathway modulated oxidative stress in late EPCs in response to AGEs.

We next investigated whether AGEs induced HMGB-1 expression in late EPCs via ROS production by using NAC to inhibit oxidative stress induced in late EPCs by AGEs. The ROS generation in late EPCs demonstrated by DCFH-DA staining was significantly increased by AGE exposure; this effect was abrogated by N-acetylcysteine (NAC) treatment (Figure 3(c)). NAC abolished the stimulatory effect of AGEs on HMGB-1 expression in late EPCs (Figure 3(d)), suggesting that AGEs induce HMGB-1 expression in late EPCs via oxidative stress.

### 3.4. Inhibition of HMGB-1 Attenuated Oxidative Stress Induced by AGEs in Late EPCs

**Figure 4:**
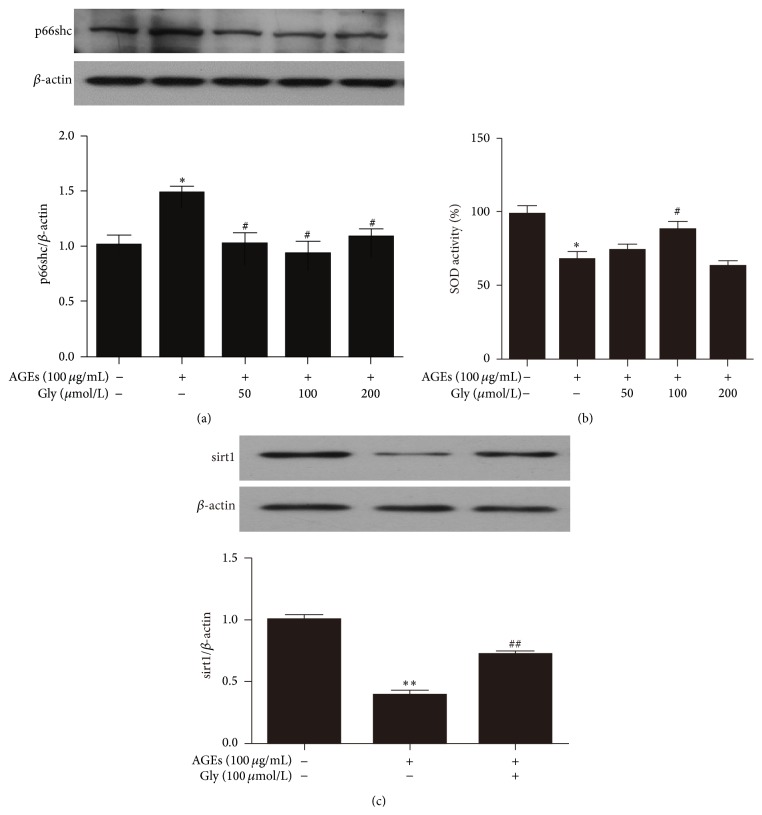
Glycyrrhizin normalized the dysregulated p66shc expression in AGEs treated late EPCs and the dysregulated SOD activity in cell culture supernatants. (a) The increased p66shc expression induced by AGEs was abrogated by glycyrrhizin treatment. (b) Treatment of late EPCs with glycyrrhizin reversed the decreased SOD activity induced by AGEs. (c) Glycyrrhizin attenuated the AGE-induced decrease in sirt1 expression in late EPCs. ^*∗*^ *P* < 0.05 versus control group, ^*∗∗*^ *P* < 0.01 versus control group, ^#^ *P* < 0.05 versus AGEs group, and ^##^ *P* < 0.01 versus AGE group, *n* = 3. Gly: glycyrrhizin.

Glycyrrhizin was used to block the stimulation of HMGB-1 to determine its role in AGE-induced oxidative stress detected by intracellular p66shc expression and superoxide dismutase (SOD) activity in cell culture supernatants. Treatment of late EPCs with glycyrrhizin abrogated AGE-induced upregulation of p66shc expression (Figure 4(a)). Furthermore, the decreased SOD activity induced by AGEs was reversed by the glycyrrhizin administration (Figure 4(b)). Similar to p66shc, AGE-induced dysregulation of sirt1 expression was suppressed by the inhibition of HMGB-1 (Figure 4(c)).

## 4. Discussion

**Figure 5:**
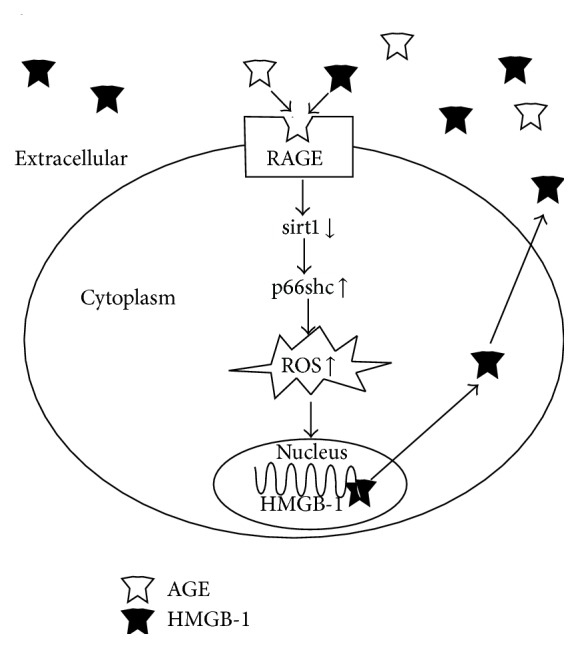
Hypothetical diagram of HMGB-1-mediated enhancement of oxidative stress induced in late EPCs by AGEs. AGEs induced ROS production via the sirt1/p66shc pathway and then promoted HMGB-1 release from the late EPCs. Extracellular HMGB-1 augmented ROS production via binding to RAGE and signaling through the sirt1/p66shc pathway.

In the present study, we demonstrate for the first time that AGEs induce HMGB-1 expression in late EPCs via sirt1/p66shc-mediated oxidative stress and that the inhibition of HMGB-1 attenuates sirt1/p66shc pathway activity and oxidative stress in late EPCs exposed to AGEs. Thus, we conclude that HMGB-1 may amplify sirt1/p66shc pathway signaling and oxidative stress reactions in late EPCs induced by AGEs (Figure 5).

HMGB-1 is a nuclear DNA-binding protein that regulates gene transcription and maintains the nucleosome structure. Under stress conditions, HMGB-1 is released from the cell and functions as a multifunctional cytokine that contributes to various pathophysiological processes.

Accumulating clinical and experimental evidence demonstrates the presence of elevated serum HMGB-1 in diabetic patients and increased HMGB-1 expression in diabetic animals [18–20]. Furthermore, a recent study showed that plasma HMGB-1 levels were increased in Chinese subjects with pure type 2 DM [21]. In accordance with previous studies, the current investigation demonstrated that serum HMGB-1 levels were significantly higher in STZ-induced diabetic mice compared with their nondiabetic counterparts. It is worth mentioning that increased HMGB-1 expression was also observed in bone marrow-derived monocytes from diabetic mice. It can be speculated that EPCs from diabetic mice also exhibited higher HMGB-1 expression because the EPCs were predominantly mobilized from bone marrow. Although we did not detect expression of HMGB-1 in EPCs from diabetic mice directly, our in vitro studies demonstrated that HMGB-1 expression increased significantly and dose dependently in late EPCs following the exposure to AGEs and was accompanied by an increase in the levels detected in cell culture supernatants. This is in accordance with previous reports that hyperglycemia induced HMGB-1 expression in endothelial cells, vascular smooth muscle cells, and cardiomyocytes [22–24]. In addition, the underlying mechanism by which diabetes induces HMGB-1 was investigated in this study.

Oxidative stress has been reported to be involved in active HMGB-1 secretion [15, 25], and HMGB-1 in animal models is inhibited by antioxidants such as resveratrol [19, 26]. As oxidative stress is a central step leading to EPC dysfunction in diabetes and thus contributes to cardiovascular impairment [1, 27], we hypothesize that diabetes induces the upregulation and release of HMGB-1 in late EPCs via oxidative stress. As shown in Figure 3, production of both HMGB-1 and ROS induced by AGEs in late EPCs was partially abolished by treatment with NAC, suggesting that oxidative stress is involved in AGE-induced HMGB-1 expression and that HMGB-1 production occurs downstream of ROS production in late EPCs stimulated by AGEs.

RAGE belongs to an immunoglobulin superfamily of cell surface molecules with the capacity to bind to a number of ligands such as AGEs, HMGB-1, amyloid fibrils, and S-100 proteins and is involved in a variety of cellular functions [28]. It is generally accepted that RAGE activation contributes to oxidative stress in diabetes [13, 29]. HMGB-1 is a well-known non-AGE ligand for RAGE and can induce endothelial dysfunction [30]; therefore, HMGB-1 may cause increased ROS generation in late EPCs.

Oxidative stress is defined as an imbalance between ROS generation and antioxidant defenses. The adaptor protein, p66shc, promotes ROS production [31, 32], while SOD functions as an intracellular enzymatic antioxidant by scavenging excess oxygen-free radicals. Thus, p66shc expression in late EPCs and SOD activity in cell culture supernatants were determined to evaluate the effects of glycyrrhizin on oxidative stress induced in late EPCs by AGEs. In accordance with the findings of others [33, 34], the present study showed dysregulation of p66shc expression and SOD activity in AGEs-stimulated late EPCs. Furthermore, the changes in p66shc and SOD activity were partly normalized by inhibiting HMGB-1 with glycyrrhizin, suggesting that HMGB-1 mediates oxidative stress induced in late EPCs by AGEs. In accordance with this speculation, Feng demonstrated that HMGB-1 mediated endothelial activation by oxidative stress responses via the RAGE pathway [30]. Furthermore, Zhang recently showed that HMGB-1 induced a marked increase in intracellular ROS in cardiomyocytes [16]. All these observations indicate that HMGB-1 stimulates oxidative stress in a variety of cell types.

Sirt1, a mammalian homologue of sir2, was identified as a key mediator of various diseases. Accumulating evidence suggests that sirt1 promotes cellular resistance to oxidative stress associated with diabetes. In the current investigation, we demonstrated that AGE treatment significantly decreased sirt1 expression in late EPCs, while resveratrol, an activator of sirt1, normalized the downregulated sirt1 expression. Furthermore, resveratrol treatment inhibited AGE-induced p66shc expression in late EPCs, although the effect was lost when AGE-stimulated late EPCs were pretreated with the sirt1 antagonist, Ex527. In accordance with previous observations that p66shc modulates oxidative stress in EPCs in response to high glucose [33], our results confirm that AGEs induce increased p66shc expression in late EPCs via downregulation of sirt1.

## 5. Conclusions

The results of the present study, together with the previous findings, indicate that HMGB-1 is not only an effector of oxidative stress, but also an inducer of oxidative stress in diabetes-induced late EPCs. In addition, targeting HMGB-1 with glycyrrhizin to inhibit oxidative stress is implicated as an attractive therapeutic strategy for impaired function of late EPCs in DM.

## References

[1] Kim K.-A., Shin Y.-J., Kim J.-H. (2012). Dysfunction of endothelial progenitor cells under diabetic conditions and its underlying mechanisms. *Archives of Pharmacal Research*.

[2] Tousoulis D., Papageorgiou N., Androulakis E. (2013). Diabetes mellitus-associated vascular impairment: novel circulating biomarkers and therapeutic approaches. *Journal of the American College of Cardiology*.

[3] Alev C., Ii M., Asahara T. (2011). Endothelial progenitor cells: a novel tool for the therapy of ischemic diseases. *Antioxidants and Redox Signaling*.

[4] Georgescu A., Alexandru N., Constantinescu A., Titorencu I., Popov D. (2011). The promise of EPC-based therapies on vascular dysfunction in diabetes. *European Journal of Pharmacology*.

[5] Loomans C. J. M., De Koning E. J. P., Staal F. J. T. (2004). Endothelial progenitor cell dysfunction: a novel concept in the pathogenesis of vascular complications of type 1 diabetes. *Diabetes*.

[6] António N., Fernandes R., Soares A. (2014). Reduced levels of circulating endothelial progenitor cells in acute myocardial infarction patients with diabetes or pre-diabetes: accompanying the glycemic continuum. *Cardiovascular Diabetology*.

[7] Maiese K. (2015). New insights for oxidative stress and diabetes mellitus. *Oxidative Medicine and Cellular Longevity*.

[8] Loomans C. J. M., De Koning E. J. P., Staal F. J. T., Rabelink T. J., Van Zonneveld A.-J. (2005). Endothelial progenitor cell dysfunction in type 1 diabetes: another consequence of oxidative stress?. *Antioxidants and Redox Signaling*.

[9] Fadini G. P., Losordo D., Dimmeler S. (2012). Critical reevaluation of endothelial progenitor cell phenotypes for therapeutic and diagnostic use. *Circulation Research*.

[10] Bodiga V. L., Eda S. R., Bodiga S. (2014). Advanced glycation end products: role in pathology of diabetic cardiomyopathy. *Heart Failure Reviews*.

[11] Cooper M. E. (2004). Importance of advanced glycation end products in diabetes-associated cardiovascular and renal disease. *American Journal of Hypertension*.

[12] Hanssen N. M. J., Beulens J. W. J., Van Dieren S. (2015). Plasma advanced glycation end products are associated with incident cardiovascularevents in individuals with type 2 diabetes: a Case-Cohort study with a median follow-Up of 10 years (EPIC-NL). *Diabetes*.

[13] Chen J., Song M., Yu S. (2010). Advanced glycation endproducts alter functions and promote apoptosis in endothelial progenitor cells through receptor for advanced glycation endproducts mediate overpression of cell oxidant stress. *Molecular and Cellular Biochemistry*.

[14] Chen Q., Dong L., Wang L., Kang L., Xu B. (2009). Advanced glycation end products impair function of late endothelial progenitor cells through effects on protein kinase Akt and cyclooxygenase-2. *Biochemical and Biophysical Research Communications*.

[15] Yu Y., Tang D., Kang R. (2015). Oxidative stress-mediated HMGB1 biology. *Frontiers in Physiology*.

[16] Zhang C., Mo M., Ding W. (2014). High-mobility group box 1 (HMGB1) impaired cardiac excitation-contraction coupling by enhancing the sarcoplasmic reticulum (SR) Ca^2+^ leak through TLR4-ROS signaling in cardiomyocytes. *Journal of Molecular and Cellular Cardiology*.

[17] Hermansen S. E., Lund T., Kalstad T., Ytrehus K., Myrmel T. (2011). Adrenomedullin augments the angiogenic potential of late outgrowth endothelial progenitor cells. *The American Journal of Physiology—Cell Physiology*.

[18] Yamashita A., Nishihira K., Matsuura Y. (2012). Paucity of CD34-positive cells and increased expression of high-mobility group box 1 in coronary thrombus with type 2 diabetes mellitus. *Atherosclerosis*.

[19] Delucchi F., Berni R., Frati C. (2012). Resveratrol treatment reduces cardiac progenitor cell dysfunction and prevents morpho-functional ventricular remodeling in type-1 diabetic rats. *PLoS ONE*.

[20] Škrha J., Kalousová M., Švarcová J. (2012). Relationship of soluble RAGE and RAGE ligands HMGB1 and EN-RAGE to endothelial dysfunction in type 1 and type 2 diabetes mellitus. *Experimental and Clinical Endocrinology and Diabetes*.

[21] Wang H., Qu H., Deng H. (2015). Plasma HMGB-1 levels in subjects with obesity and type 2 diabetes: a cross-sectional study in China. *PLOS ONE*.

[22] Yao D., Brownlee M. (2010). Hyperglycemia-induced reactive oxygen species increase expression of the receptor for advanced glycation end products (RAGE) and RAGE ligands. *Diabetes*.

[23] Wang Y., Shan J., Yang W., Zheng H., Xue S. (2013). High mobility group box 1 (HMGB1) mediates high-glucose-induced calcification in vascular smooth muscle cells of saphenous veins. *Inflammation*.

[24] Wang W.-K., Lu Q.-H., Zhang J.-N. (2015). HMGB1 mediates hyperglycaemia-induced cardiomyocyte apoptosis via ERK/Ets-1 signalling pathway. *Journal of Cellular and Molecular Medicine*.

[25] Maugeri N., Rovere-Querini P., Baldini M. (2014). Oxidative stress elicits platelet/leukocyte inflammatory interactions via HMGB1: a candidate for microvessel injury in sytemic sclerosis. *Antioxidants & Redox Signaling*.

[26] Xu W., Lu Y., Yao J. (2014). Novel role of resveratrol: suppression of high-mobility group protein box 1 nucleocytoplasmic translocation by the upregulation of sirtuin 1 in sepsis-induced liver injury. *Shock*.

[27] Hamed S., Brenner B., Aharon A., Daoud D., Roguin A. (2009). Nitric oxide and superoxide dismutase modulate endothelial progenitor cell function in type 2 diabetes mellitus. *Cardiovascular Diabetology*.

[28] Kalousová M., Zima T. (2014). AGEs and RAGE—advanced glycation end-products and their receptor in questions and answers. *Vnitrni Lekarstvi*.

[29] Chen J., Huang L., Song M., Yu S., Gao P., Jing J. (2009). C-reactive protein upregulates receptor for advanced glycation end products expression and alters antioxidant defenses in rat endothelial progenitor cells. *Journal of Cardiovascular Pharmacology*.

[30] Feng L., Zhu M., Zhang M. (2013). Amelioration of compound 4,4′-diphenylmethane-bis(methyl)carbamate on high mobility group box1-mediated inflammation and oxidant stress responses in human umbilical vein endothelial cells via RAGE/ERK1/2/NF-*κ*B pathway. *International Immunopharmacology*.

[31] Vikram A., Kim Y.-R., Kumar S. (2014). Canonical Wnt signaling induces vascular endothelial dysfunction via p66Shc-regulated reactive oxygen species. *Arteriosclerosis, Thrombosis, and Vascular Biology*.

[32] Zhou S., Chen H.-Z., Wan Y.-Z. (2011). Repression of P66Shc expression by SIRT1 contributes to the prevention of hyperglycemia-induced endothelial dysfunction. *Circulation Research*.

[33] Di Stefano V., Cencioni C., Zaccagnini G., Magenta A., Capogrossi M. C., Martelli F. (2009). P66ShcA modulates oxidative stress and survival of endothelial progenitor cells in response to high glucose. *Cardiovascular Research*.

[34] Li H., Zhang X., Guan X. (2012). Advanced glycation end products impair the migration, adhesion and secretion potentials of late endothelial progenitor cells. *Cardiovascular Diabetology*.

